# The Relationship Between Humor Styles and Life-Meaning Orientations in Students

**DOI:** 10.1192/j.eurpsy.2026.11834

**Published:** 2026-07-31

**Authors:** A. Akhmetzyanova, T. Artemyeva, I. Artemyeva

**Affiliations:** 1 Department of Psychology and Pedagogy of Special Education, Kazan Federal University, Kazan; 2 Institute of Pedagogy and Psychology, Moscow State Pedagogical University, Moscow, Russian Federation

## Abstract

**Introduction:**

In adolescence and early adulthood, there is an active formation of personal and professional orientations among young people as they begin to become aware of their values, goals, and sense of life meaning. The system of value and life-meaning orientations in students of higher education institutions enables them to manage their lives, take responsibility for the consequences of their actions, establish social relationships with others, and interact harmoniously with them through the use of humor.

**Objectives:**

to identify the relationship between humor styles and life-meaning orientations in undergraduate students.

**Methods:**

The study involved 208 undergraduate students (15 male and 193 female). The following instruments were used: *Purpose-in-Life Test* by J. Crumbaugh and L. Maholic, adapted by D. A. Leontiev; *Diagnostics of Life Goals: Brief Version of the Aspiration Index Questionnaire* by T. O. Gordeeva, O. A. Sycheva, and V. A. Egorova; and the Russian-language adaptation of R. Martin’s *Humor Styles Questionnaire.* Pearson’s correlation analysis was applied for statistical data processing.

**Results:**

The study revealed that affiliative humor, characterized by cheerfulness, predominance of positive emotions, and oriented toward maintaining and establishing interpersonal relationships, is associated with social life-meaning indicators: *Community* (r = .207, p < .01) and *Fame* (r = .183, p < .01). Self-enhancing humor, used by students primarily for the regulation of their inner life, is positively associated with all indicators of life-meaning orientations: *Goals* (r = .243, p < .01), *Process* (r = .255, p < .01), *Result* (r = .300, p < .01), *Locus of Self* (r = .253, p < .01), and *Locus of Life* (r = .216, p < .01), which indicates the internal regulatory role of humor in the formation and realization of students’ life-meaning orientations. Self-enhancing humor is also positively associated with *Community* (r = .274, p < .01), contributing to the establishment of positive relationships in social interaction.

Negative correlations were found between aggressive humor and all indicators of students’ life-meaning orientations: *Goals* (r = –.281, p < .01), *Process* (r = –.255, p < .01), *Result* (r = –.336, p < .01), *Locus of Self* (r = –.222, p < .01), and *Locus of Life* (r = –.335, p < .01). Aggressive humor does not facilitate the establishment of social relationships in the community (r = –.240, p < .01); however, according to respondents, this type of humor is perceived as capable of influencing others (r = .181, p < .01).

**Conclusions:**

The results of the study make it possible to develop programs and training sessions aimed at optimizing the educational process in higher education institutions and fostering the formation of life goals among students.

**Disclosure of Interest:**

None Declared

